# Instructional design and educational satisfaction for virtual environment simulation in undergraduate nursing education: the mediating effect of learning immersion

**DOI:** 10.1186/s12909-022-03728-6

**Published:** 2022-09-12

**Authors:** So Young Park, Jung-Hee Kim

**Affiliations:** College of Nursing, The Catholic University of Korea, 222 Banpo-daero Seocho-gu, Seoul, 06591 South Korea

**Keywords:** Nursing education, Nursing students, Instructional design, Learning immersion, Educational satisfaction, Path analysis

## Abstract

**Background:**

Schools were closed after the onset of COVID-19, with non-face-to-face practices or distance education in nursing education replacing video learning or simulation classes in nursing education clinical practicum. This led to an increase in interest in virtual environment simulation education. While technology-based teaching methods might feel new and intriguing to learners, it is necessary to evaluate learner satisfaction with such an educational method beyond its novelty value. Therefore, this study examined the mediating effect of learning immersion on the relationships between instructional design and educational satisfaction, for virtual environment simulation.

**Methods:**

A descriptive cross-sectional research design was used. The study sample included students in the third or fourth year of the nursing curriculum in South Korea. The participants were 164 nursing students, who had an experience with virtual environment simulation practices during the past year, prior to September 2021. Data were collected using an online questionnaire. The questionnaire addressed the characteristics of nursing students, instructional design, learning immersion, and educational satisfaction. The collected data were analyzed using path analysis.

**Results:**

The indirect effect of the path between instructional design and educational satisfaction, mediated through learning immersion in virtual environment simulation was found to be significant. Furthermore, the direct path was also statistically significant.

**Conclusion:**

Educational content, based on virtual environment simulation, should be implemented based on instructional design. It is necessary to recognize the importance of instructional design that can promote learning immersion in virtual environment simulation, as well as to prepare consistent standards for such design.

## Background

Nursing education provides students with the knowledge and skills they will require as professional nurses [1, 2]. Simulation-based learning has been proposed as an educational strategy that can replace the clinical practicum because it makes it possible to construct a controlled clinical environment and perform repeated practices without impacting patient safety [3, 4].

With the recent developments in technology, research has been conducted on the effectiveness of virtual environment (VE) simulation education using technology as a strategy for future clinical practicum [5, 6]. The limitations of tools used in learning are addressed through convergence technologies such as augmented reality and virtual reality. Such education will bring innovation that expands and evolves not only in the school context but also in daily life [7, 8]. Schools were closed owing to the onset of COVID-19, with non-face-to-face practice or distance education replacing video learning or simulation classes in the nursing education clinical practicum [9], leading to a greater interest in VE simulation education.

VE simulation education has been used in surgical skill disciplines and anatomy classes in medical schools [10, 11]. In nursing colleges, VE simulation has been widely adopted in various skill disciplines such as those related to sterile techniques, medication administration, urinary catheterization, and nursing care for neonatal patients and patients with chronic obstructive lung disease [12–15].

A VE refers to an environment where two-dimensional (2D), or three-dimensional (3D) images were implemented with a computer, mobile device, or virtual reality/augmented reality/mixed reality device [16], and does not include multiple characters or participants [17]. In nursing education, VE simulation consists of two types: virtual reality simulation and virtual simulation [6, 18]. Virtual reality simulation involves interactions through 3D images with the use of a computer keyboard, mouse, motion sensors, and haptic devices [19]. Virtual simulation is where a user interacts with an environment presented on the computer screen [19]. VE simulation operates in the same way as the existing simulations and monitors students’ reactions and provides feedback, but it is implemented in a VE [20]. Students of the current generation have the ability to quickly adapt to digital devices and learn by using them [21]. VE educational content that suits the characteristics of these learners, can help them learn comfortably in a safe environment [22], and develop empathy through the simulation of direct experiences [23].

According to the Jeffries simulation framework, the five key components of simulation are participant, facilitator, educational practices, simulation design characteristics, and outcomes [24]. The instructional design indicates the extent to which learners perceive the learning objectives, planning, fidelity, complexity, cues, and debriefing [25, 26]. Outcomes include knowledge, skill performance, learner satisfaction, critical thinking, and self-confidence [24]. Educational satisfaction refers to the evaluation of the learner’s educational experiences, and learner satisfaction should be considered when using new teaching methods [27]. Factors affecting educational satisfaction include self-directed learning readiness, professor-student interactions, learning immersion [28], course content [29], and course design [30].

While technology-based teaching methods might seem new and intriguing to learners, it is necessary to evaluate learner satisfaction with such an educational method beyond its novelty value. Learning outcomes are affected by educational satisfaction, which plays a significant role in determining behavior and intention [31, 32].

Well-designed simulation education can increase learning immersion and learning outcomes [26, 33]. In simulation education, systematic instructional design can efficiently execute the curriculum [34], motivate learners to actively participate, and enable them to focus [35]. In distance education, learning immersion through psychological mechanisms is an important aspect of instructional design [26].

Immersion is a positive experience that an individual feels, by maximizing their concentration on the activity [36], and learning immersion affects educational satisfaction [37, 38]. Learning immersion induces concentration and participation in the acquisition of knowledge, which enhances learning outcomes. This emphasizes the importance of the immersion experience, in creating high learning outcomes by improving intrinsic motivation in distance education, which depends on learners’ self-direction [35]. Learners’ immersion affects satisfaction and perception, even in VE education contexts [39]. Although several studies have been conducted on satisfaction evaluation for simulation-based learning [6, 7, 9, 20, 26, 33, 40, 41], there is a lack of research on the satisfaction dimesion related to VE education. Consequently, there is limited information to support designing and applying VE educational content for instructors unfamiliar with the use of VE simulations.

This study examined the mediating effect of learning immersion on the relationships between instructional design, and educational satisfaction in VE simulation, using path analysis. The study hypotheses are as follows:

- H1: Instructional design in VE simulation has a direct effect on learning immersion.
- H2: Learning immersion in VE simulation has a direct effect on educational satisfaction.
- H3: Learning immersion in VE simulation has a mediating effect on the relationship between instructional design and educational satisfaction.

## Methods

### Design

This descriptive cross-sectional study examined the effect of instructional design in VE simulation, as perceived by nursing students on educational satisfaction through learning immersion.

### Participants

The participants were nursing students, who were experienced in VE simulation practices, during the past year, in a nursing college in South Korea. The inclusion criteria were as follows: (1) students in the third or fourth year of the nursing curriculum; (2) students who understood the purpose of this study; and (3) students who expressed their intention to participate voluntarily. The exclusion criterion is students who had experienced only nursing skills-based VE simulations without a scenario.

To conduct a path analysis, the recommended adequate sample size is 20 times the number of measurement variables [42]. Hence, three observation variables should have at least 60 samples. In this study, 164 samples were used for the final analysis.

### Data collection

The study received ethical approval from the Catholic University of Korea Research Ethics Committee (No. MC21QESI0092), and informed consent was obtained from all subjects. Data were collected for September 2021, using online questionnaires. Recruitment documents, containing the study title, objectives, method, participant criteria, and participation benefits and risks, were distributed across online nursing college communities in South Korea, informing the participants about the study. Based on this information, students could decide to participate in the study by clicking on the link to the online questionnaire. A total of 180 respondents were included in this study, 16 of whom had incomplete data, and the remaining 164 respondents were analyzed.

### Instruments

#### Instructional design

The instructional design was measured using the Korean version [26] of the Simulation Design Scale, developed by National League for Nursing [43]. This scale consists of 21 items, measured using five subscales, including educational goals and content, support, problem-solving, feedback, and fidelity. Each item is measured on a five-point Likert scale, ranging from “strongly disagree” to “strongly agree.” Higher scores indicated superior instructional design. Cronbach’s α was 0.92 in this study.

#### Learning immersion

Learning immersion was measured using the Learning Immersion in Simulation Scale, which was originally developed by Ko [44]. There were a total of 16 items measured using four subscales: cognitive elaboration, presence, concentration, and autotelic experience. Each item is measured on a five-point Likert scale, ranging from “strongly disagree” to “strongly agree”. Higher scores indicated higher learning immersion. In this study, Cronbach’s α was 0.90.

#### Educational satisfaction

Educational satisfaction was measured using the Educational Satisfaction Scale in Simulation for Nursing Students, by Kim and Heo [45]. The scale comprises 16 items measured using three subscales: learning content, situational competency, and emotional response. These were measured using a five-point Likert scale, ranging from “strongly disagree” (one point) to “strongly agree” (five points). Higher scores indicate higher levels of educational satisfaction. Cronbach’s α was 0.78 in this study.

### Data analysis

Correlations with the variables were processed using Pearson’s correlation coefficient, to test for multicollinearity. The correlation coefficient ranged from 0 to 1, with values of 0.80 or less [46], and the variance inflation factor (VIF) values were less than 10 [47].

Structural relationships between variables were identified by analyzing indirect effects using the model. The sizes of the direct effect, indirect effect, and total effect were calculated by bootstrapping, to confirm the significance of the mediating effect. The collected data were analyzed using IBM SPSS for Windows, version 26.0 (IBM corp., Armonk, NY, USA) and AMOS, version 26.0 (IBM Corp., Armonk, NY, USA).

## Results

### General characteristics

The participants were 56 (34.1%) junior and 108 (65.9%) senior nursing students. Of these, 19 (11.6%) were men and 145 (88.4%) were women. The average age of the participants was 23.03 years (SD = 2.71). The average experience frequency of VE simulation was 6.23 (SD = 4.63), with 97% of them having experienced it five times or less. The VE simulation product experienced the most was vSIM® for Nursing, with 153 participants (93.9%), and the discipline in which VE simulation was experienced the most was adult health nursing, with 144 participants (87.8%) (Table 1).

**Table 1:** General characteristic of participants (*N* = 164)

Variables		N(%)
Year	Junior	56(34.1)
	Senior	108(65.9)
Gender	Male	19(11.6)
	Female	145(88.4)
Age	20 ~ 22	89(54.3)
	23 ~ 25	57(34.8)
	Above 26	18(11.0)
Grade	High	52(31.7)
	Medium	100(61.0)
	Low	12(7.3)
Experience of VE^a^	~ 5	93(56.7)
	6 ~ 10	59(36.0)
	Above 11	12(7.3)
Products of VE^a^	vSIM® for Nursing	153(93.3)
	HoloPatient	30(18.3)
	MUVE^b^	7(4.3)
	Second Life	1(0.6)
	Unreal Engine	1(0.6)
	Others	7(4.3)
Nursing subjects of VE^a^	Adult health	144(87.8)
	Pediatric	79(48.2)
	Mental	83(50.6)
	Maternal	85(51.8)
	Community health	62(37.8)
	Others	4(2.4)

^a^*VE* Virtual environment, ^b^*MUVE* Multi-User Virtual Environments

### Descriptive statistics

The average score for instructional design was 79.30 (SD = 10.45); the average score for learning immersion was 56.37 (SD = 8.49); and the average score for educational satisfaction was 57.26 (SD = 6.53).

### Multicollinearity

Correlations were calculated to determine whether there was multicollinearity between the variables. The correlations are presented in Table 2. The three variables had a significant correlation; the correlation coefficient did not exceed 0.80, and the multicollinearity VIF was 2.507, which was less than 10. Thus, multicollinearity was not found.

**Table 2:** Variables correlations (N = 164)

Variables	Instructional design	Learning immersion
Learning immersion	.775 (<.001)	–
Educational satisfaction	.605 (<.001)	.641 (<.001)

### Hypotheses testing

**Fig. 1:**
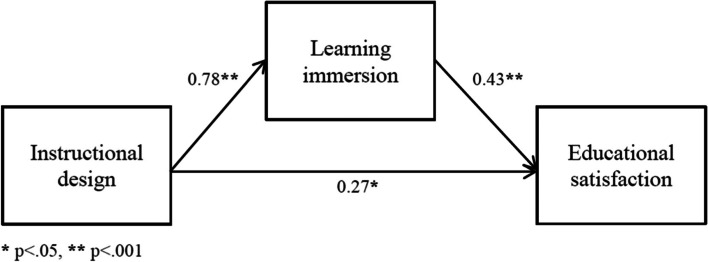
Path model

The hypothesized model, with standardized path coefficients and direct effect estimates between variables in the path model, are displayed in Table 3. The direct path from instructional design to learning immersion in VE simulation (β = .775, *p* < .001), and from learning immersion to educational satisfaction in VE simulation (β = .431, p < .001), were statistically significant. The direct path from instructional design to educational satisfaction in VE simulation (β = .271, *p* = .004), was also statistically significant (Fig. 1).

**Table 3:** Path coefficients of the proposed model

Path			B	β	S.E.	C.R.	p
Instructional design	→	Learning Immersion	0.630	0.775	0.040	15.675	<.001
Instructional design	→	Educational Satisfaction	0.169	0.271	0.058	2.917	.004
Learning Immersion	→	Educational Satisfaction	0.332	0.431	0.071	4.641	<.001

Covariate decomposition of the final model was performed to examine the direct, indirect, and total effects between each pair of variables (Table 4). The indirect effect of the path from instructional design to educational satisfaction with the VE simulation mediated by learning immersion (*p* = .001), was significant. Further, 60.1% of the learning immersion in VE simulation was explained by instructional design, and 44.0% of the educational satisfaction in VE simulation was explained by instructional design and learning immersion.

**Table 4:** Direct and indirect effects of variables

Predictor variables	Dependent variables	Total effect	Direct effect	Indirect effect	p	R^2^
Instructional design	Learning immersion	.775	.775		.001	.601
Instructional design	Educational Satisfaction	.605	.271	.209	.001	.552
Learning immersion		.431	.431			

## Discussion

This study investigated the effect of the instructional design of VE-based nursing education content, on educational satisfaction. The path model was constructed to reveal the direct and indirect effects in the relationship between variable pairs mediated by learning immersion. The results showed that instructional design in VE simulation affected educational satisfaction, and that learning immersion had a partial mediating effect on the relationship between instructional design and educational satisfaction in VE simulation.

It was also noted, when nursing students recognized that the instructional design was well structured in the VE simulation, it induced higher learning immersion and increased educational satisfaction. The students perceived that instructional design was well-structured in VE simulations, which promoted self-learning satisfaction [28]. The instructional design of simulations is considered important for the achievement of learning outcomes [34]. VE simulation is a situation in which interaction in real time between learners and instructors is difficult; therefore, media containing educational content that can promote interaction between instructors between them should be provided through instructional design, to increase the satisfaction of learners [28, 48]. Therefore, instructors need to plan and implement instructional design methods in VE simulations with this interaction in consideration.

The finding that instructional design in VE simulation had a direct effect on educational satisfaction, as well as an indirect effect on learning immersion as a mediating variable, indicates the importance of instructional design in VE simulation education. However, although the positive results of a meta-analysis study of VE simulation in nursing education provide supportive evidence of the applicability thereof, most studies focus only on new technologies and do not provide design-specific details [6]. Even if the instructional design in VE simulation was developed considering the needs of nursing students and their levels, the content resulted in different outcomes, depending on the learner. Therefore, instructional design in VE simulation should be more sophisticated and systematic, and not limited to the existing educational methods. Instructional design in simulation consists of role distribution, pre-briefing, simulation execution, and debriefing of learners based on clear objectives [49]. Learning objectives, support, problem-solving, feedback, and fidelity should be considered when the instructor designs a simulation [50]. In simulation education, the instructor’s role is to plan and organize the learning environment [25]. Instructors can use the following tips for teaching and evaluating clinical reasoning in simulation education: emphasizing the importance of evidence in clinical decision-making; including continuous and immediate feedback; self-assessment and formative assessment; and encouraging post-care [51]. Therefore, it is necessary to recognize the importance of instructional design in strengthening the learning immersion in VE simulation, and to prepare consistent standards for the design thereof.

In the model, the mediating variable, namely the learning immersion in VE simulation, partially mediated the relationship between instructional design and educational satisfaction. Learning immersion is an important mediating variable in the relationship between learning presence and satisfaction [52], and an important factor in the design and satisfaction of simulation education [26]. Since the instructional design of simulation promotes learners’ concentration and participation, induces immersion, and promotes positive psychology, nursing students’ educational satisfaction would be improved when the instructional design of VE simulation is well structured so that they can immerse themselves in learning. The conditions of learning environments in which learners can be immersed should be established to successfully operate simulation education [53].

VE simulation education has been developing continuously together with the internet environment and improvements in computer science technology [54]. Therefore, additional research needs to evaluate learning outcomes and satisfaction according to the instructional design of VE simulation and content based on this model. Further research is also needed to verify the effect of variables according to the type of VE simulation. In terms of the technology of VE, visual factors can effectively provide information to users [55] and affect immersion and learning outcomes [20, 56]. Since the technical characteristic of VE is different from traditional practice [57], further research should consider and compare these technical and visual aspects.

This study had several limitations. The cross-sectional design limited the interpretation of causality. Since the participants responded with a self-reporting questionnaire, biased perceptions and capabilities for desirable answers may have affected the validity of the results. The data used convenience sampling, which also limited data interpretation.

## Conclusion

VE simulation-based educational content should be implemented with high-quality instructional design. Instructors should consider this and implement factors that will promote the educational satisfaction of learners experiencing VE simulation.

Such efforts to improve the quality of educational content based on VE simulation, provide useful information to nursing faculty who design and develop teaching methods for educational programs in a new environment and can also be used as a reference for further research related to educational content based on VE simulation.

## Data Availability

The datasets used and analyzed during the current study are available on reasonable request from the corresponding author.

## Abbreviations

VE: Virtual environment
VIF: Variance inflation factor

## References

[1] Gillespie M, McFetridge B (2006). Nurse education – the role of the nurse teacher. Clin Nurs.

[2] Hall-Lord ML, Theander K, Athlin E (2013). A clinical supervision model in bachelor nursing education - purpose, content and evaluation. Nurse Educ Pract.

[3] Issenberg SB, McGaghie WC, Petrusa ER, Lee Gordon D, Scalese RJ (2005). Features and uses of high-fidelity medical simulations that lead to effective learning: a BEME systematic review [article]. Med Teach..

[4] Hoffmann RL, O'Donnell JM, Kim Y (2007). The effects of human patient simulators on basic knowledge in critical care nursing with undergraduate senior baccalaureate nursing students. Simul Healthc.

[5] Khan R, Plahouras J, Johnston BC, Scaffidi MA, Grover SC, Walsh CM (2019). Virtual reality simulation training in endoscopy: a Cochrane review and meta-analysis. Endoscopy..

[6] Chen FQ, Leng YF, Ge JF, Wang DW, Li C, Chen B (2020). Effectiveness of virtual reality in nursing education: Meta-analysis. J Med Internet Res.

[7] Huang CL, Luo YF, Yang SC, Lu CM, Chen A-S (2020). Influence of students’ learning style, sense of presence, and cognitive load on learning outcomes in an immersive virtual reality learning environment. J Educ Comput Res.

[8] Uruthiralingam U, Rea PM (2020). Augmented and virtual reality in anatomical education - a systematic review. Adv Exp Med Biol.

[9] Jeon E, Peltonen LM, Block L, Ronquillo C, Tayaben JL, Nibber R (2021). Emergency remote learning in nursing education during the COVID-19 pandemic. Stud Health Technol Inform..

[10] Sikder S, Tuwairqi K, Al-Kahtani E, Myers WG, Banerjee P (2014). Surgical simulators in cataract surgery training. Br J Ophthalmol.

[11] Moro C, Štromberga Z, Raikos A, Stirling A (2017). The effectiveness of virtual and augmented reality in health sciences and medical anatomy. Anat Sci Educ.

[12] Dubovi I, Levy ST, Dagan E (2017). Now I know how! The learning process of medication administration among nursing students with non-immersive desktop virtual reality simulation. Comput Educ.

[13] Haerling KA (2018). Cost-utility analysis of virtual and mannequin-based simulation. Simul Healthc.

[14] Butt AL, Kardong-Edgren S, Ellertson A (2018). Using game-based virtual reality with haptics for skill acquisition. Clin Simul Nurs.

[15] Yu M, Yang M, Ku B, Mann JS (2021). Effects of virtual reality simulation program regarding high-risk neonatal infection control on nursing students. Asian Nurs Res.

[16] Schwebel DC, Severson J, He Y (2017). Using smartphone technology to deliver a virtual pedestrian environment: usability and validation. Virtual Real.

[17] Chang TP, Weiner D (2016). Screen-based simulation and virtual reality for pediatric emergency medicine. Clin Ped Emerg Med.

[18] Gordon RM, McGonigle D (2018). Virtual simulation in nursing education.

[19] Lopreiato JO, Anderson M, Diaz D, Robertson J, Chang T, Downing D, et al. Healthcare simulation dictionary. Agency for Healthcare Research Qual. 2020.

[20] Tolarba JEL (2021). Virtual simulation in nursing education: a systematic review. Int J Nurs Educ.

[21] Chicca J, Shellenbarger T. Generation Z: Approaches and teaching–learning practices for nursing professional development practitioners. J Nurs Prof Dev 2018;34. 10.1097/NND.0000000000000478 30188477

[22] Pottle J (2019). Virtual reality and the transformation of medical education. Future Healthc J.

[23] Gillespie GL, Farra S, Regan SL, Brammer SV (2021). Impact of immersive virtual reality simulations for changing knowledge, attitudes, and behaviors. Nurse Educ Today.

[24] Jeffries PR (2012). Simulation in nursing education: from conceptualization to evaluation.

[25] Jeffries PR (2005). A framework for designing, implementing, and evaluating simulations used as teaching strategies in nursing. Nurs Educ Perspect.

[26] Yoo J-H, Kim Y-J (2018). Factors influencing nursing students' flow experience during simulation-based learning. Clin Simul Nurs..

[27] Elliott KM, Healy MA (2001). Key factors influencing student satisfaction related to recruitment and retention [article]. J Mark Higher Educ.

[28] Cho MK, Kim MY (2021). Factors affecting learning satisfaction in face-to-face and non-face-to-face flipped learning among nursing students. Int J Environ Res Public Health.

[29] Martín-Rodríguez Ó, Fernández-Molina JC, Montero-Alonso MÁ, González-Gómez F (2015). The main components of satisfaction with e-learning. Technol Pedagog Educ.

[30] Lee J (2014). An exploratory study of effective online learning: assessing satisfaction levels of graduate students of mathematics education associated with human and design factors of an online course. IRRODL..

[31] Waluya AI, Iqbal MA, Indradewa R (2019). How product quality, brand image, and customer satisfaction affect the purchase decisions of Indonesian automotive customers [article]. Int J Serv Econ Manag.

[32] Rodríguez-García MC, Gutiérrez-Puertas L, Granados-Gámez G, Aguilera-Manrique G, Márquez-Hernández VV (2021). The connection of the clinical learning environment and supervision of nursing students with student satisfaction and future intention to work in clinical placement hospitals. J Clin Nurs.

[33] Roh YS, Jang KI, Issenberg SB (2021). Nursing students’ perceptions of simulation design features and learning outcomes: the mediating effect of psychological safety. Collegian..

[34] Committee INACSL (2016). INACSL standards of best practice: SimulationSM simulation design. Clin Simul Nurs..

[35] Lee Y-E (2021). The effect of learning presence on learning outcomes of remote classification by university students -Focusing on the medium effect of learning immersion. J Digit Conv.

[36] Csikszentmihalyi M (2014). Play and intrinsic rewards. Flow and the foundations of positive psychology.

[37] Kim UJ, Park JH (2012). The relationships among learning presence, learning flow, and academic achievement at the cyber universities. Asian J Educ.

[38] Kim YJ, Lee SH (2021). The relationships among quality of online education, learning immersion, learning satisfaction, and academic achievement in cooking-practice subject. Sustainability..

[39] Shin DH, Biocca F, Choo H (2013). Exploring the user experience of three-dimensional virtual learning environments. Behav Inf Technol.

[40] Zapko KA, Ferranto MLG, Blasiman R, Shelestak D (2018). Evaluating best educational practices, student satisfaction, and self-confidence in simulation: a descriptive study. Nurse Educ Today.

[41] Jara Jara V, Sambuceti NC (2018). Clinical simulation in nursing: a scale to evaluate satisfaction and self-confidence in learning. Stud Health Technol Inform.

[42] Mitchell RJ (2001). Path analysis. Design and analysis of ecological experiments.

[43] National League for Nursing (2005). Descriptions of available instruments.

[44] Ko EJ. The development and validation of a learning immersion in simulation scale (publication number unpublished doctor's thesis) graduate School of Hallym University. Chuncheon; 2020. http://www.riss.kr/link?id=T15829407.

[45] Kim JY, Heo NR (2019). Development of educational satisfaction scale in simulation for nursing students. Korean Assoc Learner-Centered Curric Instr.

[46] Jobson JD (1991). Confidence regions for the mean-variance efficient set: an alternative approach to estimation risk. Rev Quant Fin Acc.

[47] Cohen P, West SG, Aiken LS (2014). Applied multiple regression/correlation analysis for the behavioral sciences.

[48] Bouhnik D, Marcus T (2006). Interaction in distance-learning courses. J Am Soc Inf Sci Technol.

[49] Jeffries PR, Rodgers B, Adamson K (2015). NLN Jeffries simulation theory: brief narrative description. Nurs Educ Perspect.

[50] Jeffries PR, Rizzolo MA (2006). Designing and implementing models for the innovative use of simulation to teach nursing care of ill adults and children: a national, multi-site, multi-method study.

[51] Posel N, McGee JB, Fleiszer DM (2015). Twelve tips to support the development of clinical reasoning skills using virtual patient cases. Med Teach.

[52] Joo YJ, Joung S, Kim NH, Choung HM (2012). Factors impacting corporate e-learners' learning flow, satisfaction, and learning persistence. IADIS International Conference on Cognition and Exploratory Learning in Digital Age, CELDA.

[53] Dieckmann P, Friis SM, Lippert A, Østergaard D (2012). Goals, success factors, and barriers for simulation-based learning: a qualitative interview study in health care [article]. Simul Gaming.

[54] Foronda CL, Alfes CM, Dev P, Kleinheksel AJ, Nelson DA, OʼDonnell, J. M., & Samosky, J. T. (2017). Virtually nursing: emerging technologies in nursing education. Nurse Educ.

[55] Durlach N, Mavor AS, Newby GB (1996). Virtual reality: scientific and technological challenges. Libr Inf Sci Res.

[56] Slater M, Wilbur S (1997). A framework for immersive virtual environments (FIVE): speculations on the role of presence in virtual environments. Presence (Camb).

[57] MacLean S, Geddes F, Kelly M, Della P (2019). Realism and presence in simulation: nursing student perceptions and learning outcomes. J Nurs Educ.

